# Ubiquitination-Mediated Regulation of Brassinosteroid Signaling

**DOI:** 10.3390/plants15152256

**Published:** 2026-07-23

**Authors:** Yonghong Xie, Qin Zhang, Juansheng Ren

**Affiliations:** 1 Crop Research Institute, Sichuan Germplasm Resource Center, Sichuan Academy of Agricultural Sciences, Chengdu 610066, China; xieyh0128@163.com (Y.X.); jeanzhang666@yeah.net (Q.Z.); 2 Sichuan Provincial Key Laboratory of Crop Germplasm Innovation and Genetic Improvement, Chengdu 610066, China; 3 Qionglai Branch of the PRC Ministry of Agriculture and Rural Affairs’ Key Laboratory of Tianfu Seed Industry Innovation, Chengdu 611530, China

**Keywords:** brassinosteroids, ubiquitination, signal transduction, protein degradation, plant hormones

## Abstract

Brassinosteroids (BRs) are essential steroid hormones that coordinate plant growth, development and adaptation to changing environments. Although BR signaling has long been viewed primarily as a phosphorylation-dependent pathway, increasing evidence shows that ubiquitination provides an additional regulatory layer that shapes the abundance, activity, subcellular distribution and turnover of key signaling components. Ubiquitin-mediated regulation operates at multiple points in the BR pathway, including receptor homeostasis at the plasma membrane, turnover of GSK3-like kinases, and stability control of the transcription factors BES1/BZR1. These processes determine not only the strength and duration of BR signaling but also its coordination with other hormonal and stress-response pathways. In this review, we discuss recent advances in ubiquitin-mediated regulation of BR signaling, focusing on receptor-level control, proteolytic regulation of core signaling components, and modulation of transcriptional outputs. We also highlight emerging links between ubiquitination, selective autophagy, deubiquitination and BR-associated stress responses and outline key questions concerning ubiquitin chain specificity, substrate recognition and conservation of these regulatory modules in crops. Defining how ubiquitination fine-tunes BR signaling will deepen our understanding of plant steroid hormone regulation and may provide new strategies for optimizing crop architecture, productivity, and stress resilience.

## 1. Introduction

Brassinosteroids (BRs) are plant-specific steroid hormones that regulate cell elongation, vascular differentiation, organ growth and environmental adaptation [1,2,3]. Over the past two decades, genetic, biochemical and cell biological studies have established a core BR signaling module [4,5,6,7]. In this pathway, extracellular BRs are perceived by the plasma membrane receptor kinase BRASSINOSTEROID INSENSITIVE 1 (BRI1) and its co-receptor BRI1-ASSOCIATED RECEPTOR KINASE 1 (BAK1), which activate a phosphorylation–dephosphorylation cascade leading to regulation of the transcription factors BRASSINAZOLE-RESISTANT 1 (BZR1) and BRI1-EMS-SUPPRESSOR 1/BZR2 (BES1, also known as BZR2) [8,9]. These transcription factors control a broad set of BR-responsive genes and thereby mediate diverse developmental and physiological outputs [10,11,12].

Brassinosteroid signaling is now increasingly recognized as more than a linear growth-promoting pathway. Instead, it functions as a context-dependent regulatory module that links growth with cell fate decisions, nutrient status and environmental responses. For example, Li et al. showed that BRs repress stomatal development in etiolated Arabidopsis cotyledons through BES1/BZR1-dependent regulation of MKK9 and FAMA, revealing a role for BR signaling in epidermal cell fate control [13,14,15]. Similarly, Al-Mamun et al. reported that BES1/BZR1 mediate BR-dependent remodeling of root system architecture under low-nitrogen conditions, indicating that BR signaling contributes to nutrient-responsive developmental plasticity [16]. These studies illustrate how BR signaling connects canonical growth regulation with more specific developmental and environmental response programs [17].

Despite substantial progress in defining the core BR signaling pathway, how BR responses are quantitatively and spatially fine-tuned across developmental and environmental contexts remains incompletely understood [18]. In recent years, ubiquitination has emerged as a central regulatory mechanism in plant hormone signaling [19,20]. Although historically viewed mainly as a degradation signal that targets proteins to the 26S proteasome, ubiquitination is now recognized to regulate a broader range of cellular processes, including endocytosis and vacuolar sorting of membrane proteins, protein stability, subcellular localization and the dynamic assembly of signaling complexes [21].

This regulatory versatility is particularly evident in the BR pathway. Ubiquitination of BRI1 promotes receptor internalization and vacuolar targeting, thereby contributing to receptor turnover and modulation of BR sensitivity [22]. Conversely, the deubiquitinating enzymes UBP12 and UBP13 stabilize BRI1 by removing ubiquitin modifications, thereby maintaining receptor abundance and signaling competence [23]. At the downstream level, UBP12/UBP13-mediated deubiquitination of BES1 enhances BR signaling and promotes plant growth, indicating that the balance between ubiquitination and deubiquitination operates at multiple nodes of the pathway [24]. Together, these findings support a model in which ubiquitination is not merely a terminal degradation signal in BR signaling but an integral regulatory layer that shapes signaling amplitude, duration and specificity. Unlike previous reviews that have primarily emphasized heavy metal and arsenic stress responses and antioxidative defense mechanisms in crops [25,26], this review focuses on brassinosteroid (BR) signaling and highlights how ubiquitination and deubiquitination events coordinately regulate multiple nodes of the pathway, thereby providing an integrated regulatory framework distinct from existing studies. Compared with the recent review by Qiu et al. (2025) [27], which mainly summarized ubiquitination-dependent regulation of core BR signaling components, this review provides a broader perspective by integrating the roles of the ubiquitination machinery (E2/E3/DUBs), ubiquitin-chain topology, and post-translational regulatory networks. In addition, we further emphasize the interface between the ubiquitin–proteasome system and selective autophagy, as well as the potential implications of ubiquitin-mediated BR regulation in crop species, particularly rice.

More broadly, ubiquitination-dependent regulation of BR signaling does not act in isolation, but is closely connected with phosphorylation, endomembrane trafficking and selective autophagy. These regulatory processes together determine the intensity, duration and context dependence of BR signaling. Although this topic has attracted increasing attention, several key issues remain unresolved. These include how different ubiquitin linkage types specify distinct protein fates, whether ubiquitin-mediated BR regulatory modules are conserved or diversified across plant species, and how ubiquitination interacts with other post-translational modifications to shape BR signaling output [28,29,30]. A systematic synthesis of current progress in this field is therefore timely [31].

In this review, we summarize recent advances in ubiquitin-mediated regulation of BR signaling, focusing on receptor homeostasis, turnover of core signaling components and control of transcriptional output. We also discuss outstanding questions related to ubiquitin chain specificity, substrate recognition, pathway integration and the application of these mechanisms in crop improvement [32]. By bringing these findings together, we aim to clarify how ubiquitination fine-tunes BR signaling dynamics and to highlight regulatory nodes that may be useful for improving crop architecture, productivity and stress resilience [33].

Here, we focus on how ubiquitin-mediated BR signaling extends from Arabidopsis-based models to crops, particularly rice, where BR-related modules regulate plant architecture, grain development, yield potential and stress adaptation. We also highlight the interplay between the ubiquitin–proteasome system and selective autophagy in BES1/BZR1 stability control, as well as the potential specificity of K48- and K63-linked ubiquitin chains in BR signaling. This integrated perspective links mechanistic understanding of BR regulation with crop improvement.

### Literature Selection and Search Strategy

A literature search was conducted using Web of Science, PubMed, Scopus, and Google Scholar to identify studies related to brassinosteroid signaling and ubiquitin-mediated regulation in plants. Keywords included “brassinosteroid signaling”, “BRI1”, “BIN2”, “BES1/BZR1”, “ubiquitination”, “E3 ligase”, and “deubiquitination”, along with crop-related terms such as rice, wheat, maize, and Arabidopsis. Peer-reviewed articles and reviews published from 2000 to 2025 were considered, with emphasis on experimentally validated studies. Non-relevant or non-experimental reports were excluded. Additional studies were identified through forward and backward citation tracking.

## 2. The Brassinosteroid Signaling Pathway

### 2.1. Brassinosteroid Biosynthesis and Metabolism

Brassinosteroids (BRs) are perceived primarily at the cell surface. In *Arabidopsis thaliana*, several enzymes involved in BR biosynthesis are localized to the endoplasmic reticulum (ER) [34,35,36]. After synthesis, BRs are thought to reach the apoplastic space, where they are recognized by the plasma membrane (PM)-localized receptor kinase BRASSINOSTEROID INSENSITIVE 1 (BRI1) [37,38] and its homologues BRI1-LIKE 1 (BRL1) and BRL3 [39,40,41]. Ligand perception by these receptor kinases initiates downstream BR signal transduction. These spatially coordinated processes, encompassing ER-associated BR biosynthesis, apoplastic ligand availability and plasma membrane-based receptor activation, provide a mechanistic framework for local modulation of BR signaling output [42].

**Figure 1:**
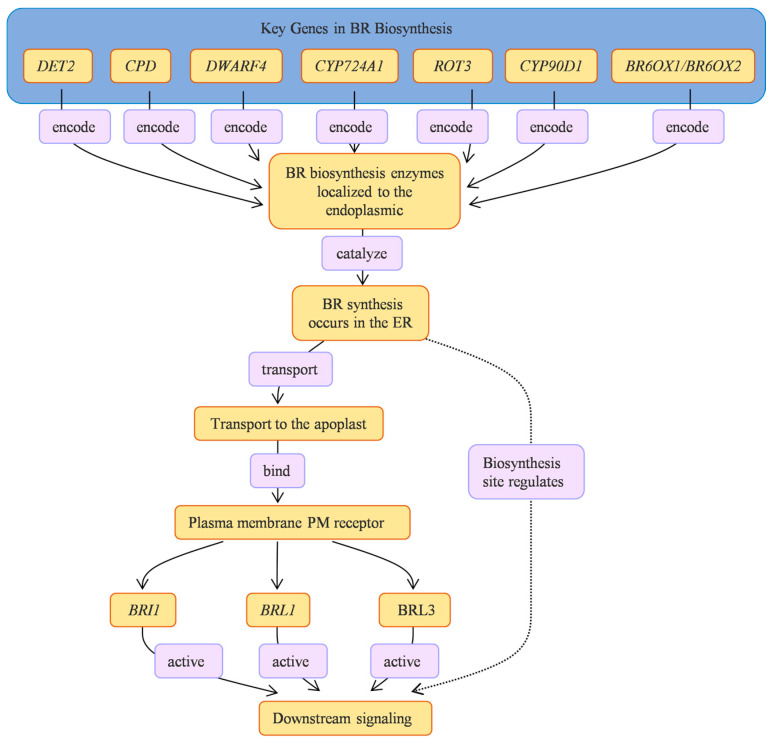
Simplified schematic of brassinosteroid (BR) biosynthesis and signaling. BR 2. *CPD*, *DWARF4*, *CYP724A1*, *ROT3*, *CYP90D1*, *BR6OX1/BR6OX2* are localized in the endoplasmic reticulum (ER), where they catalyze BR production. BRs are then transported to the apoplast and perceived by plasma membrane-localized receptors (*BRI1*, *BRL1*, *BRL3*), initiating downstream signaling cascades. Numbers indicate corresponding references.

Several key genes involved in BR biosynthesis have been identified in *Arabidopsis*, including *DET2* [43], *CPD* [44], *DWARF4* [45], *CYP724A1* [46], *ROT3* [47], *CYP90D1* [48], *BR6OX1* and *BR6OX2* [49,50] (Figure 1). The encoded enzymes are predominantly associated with the endoplasmic reticulum (ER) membrane system, where they catalyse hydroxylation, reduction and oxidation reactions required for BR production. Within the ER, BR-biosynthetic enzymes are proposed to function as multienzyme assemblies or metabolic modules that facilitate substrate channelling and support the biosynthesis of brassinolide (BL) and related BR metabolites through branched biosynthetic routes [51]. In this context, the ER acts as a central site for BR biosynthesis and an important cellular platform for controlling BR metabolic flux and downstream signaling output.

### 2.2. BR Signal Transduction Mechanism

In the model plant *Arabidopsis thaliana*, BR signaling from cell-surface perception to transcriptional regulation has been extensively characterized. In rice, a model monocot crop, BR signaling can be broadly described through two interconnected regulatory routes [52]. The first is the canonical BRI1 receptor kinase-dependent pathway. In this pathway, BRs are perceived by the plasma membrane-localized receptor kinase BRI1 and its co-receptor BAK1, leading to activation of downstream signaling cascades. GSK3-like kinases, particularly OsGSK2 in rice, act as central negative regulators by controlling the phosphorylation status and activity of BES1/BZR1-type transcription factors [53,54]. After dephosphorylation, these transcription factors accumulate in the nucleus and bind BR-responsive promoters to regulate gene expression. In *Arabidopsis*, the dominant gain-of-function mutants *bzr1-1D* and *bes1-D* stabilize BZR1 and BES1, respectively, and suppress several phenotypes of BR-deficient or BR-insensitive mutants, underscoring the central role of these transcription factors in BR signaling [55,56].

In addition to the canonical pathway, rice contains a non-canonical BR signaling module mediated by the heterotrimeric G-protein α subunit D1/RGA1. D1/RGA1 interacts with the U-box E3 ubiquitin ligase TUD1, forming a distinct regulatory module that contributes to BR responses. This D1/RGA1-TUD1 pathway intersects with the BRI1-dependent pathway and, together with canonical BR signaling components, regulates BR-mediated growth and developmental processes in rice [57].

A complete signaling pathway generally comprises three core processes: signal perception, signal transduction and downstream response. In rice, BR perception occurs at the plasma membrane, where BRs are recognized by the receptor kinase OsBRI1. Ligand binding to the extracellular domain of OsBRI1 activates its kinase activity and promotes recruitment of the co-receptor kinase OsBAK1, leading to formation of an active receptor complex that initiates downstream signaling. Activation of the receptor complex results in phosphorylation-dependent inactivation of OsGSK1 and OsGSK12, thereby relieving their repression of downstream signaling components. OsGSK2, another key GSK3-like kinase, negatively regulates BR responses by phosphorylating and repressing the activity of DWARF AND LOW-TILLERING (DLT), a GRAS-family transcription factor. In addition, DLT expression is negatively regulated by OsBZR1, a central transcription factor in the BR pathway. These interactions establish a multilayered regulatory network in which receptor activation, GSK3-like kinase inhibition and transcriptional feedback collectively fine-tune BR signal transduction in rice [54].

**Figure 2:**
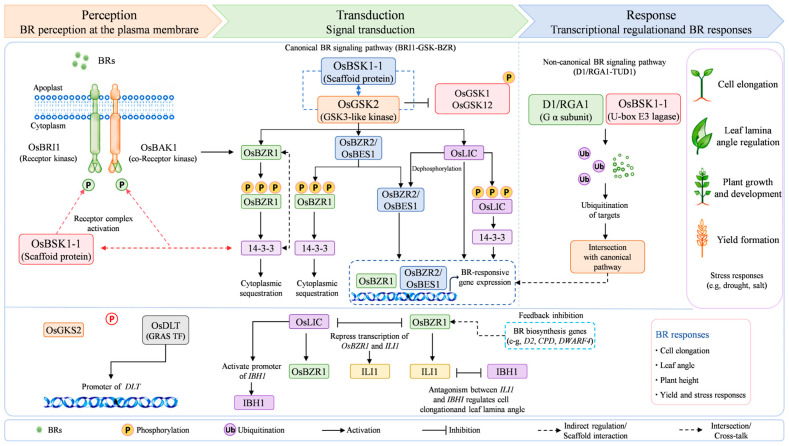
Schematic model of canonical and non-canonical brassinosteroid (BR) signaling pathways in rice. BR is perceived by the OsBRI1–OsBAK1 receptor complex, activating downstream signaling via OsBSK1-1. OsGSK2 phosphorylates OsBZR1, OsBZR2, and OsLIC, promoting their cytoplasmic retention via 14-3-3 proteins. Dephosphorylation enables nuclear accumulation of OsBZR1/OsBZR2 to activate BR-responsive genes and feedback regulation of BR biosynthesis. OsLIC negatively regulates BR signaling through transcriptional control of OsBZR1, ILI1, and IBH1. The D1/RGA1–TUD1 module provides a non-canonical ubiquitination-dependent pathway that converges with canonical BR signaling to regulate growth, architecture, and stress responses.

How BR-regulated phosphorylation controls the activity of BES1/BZR1 is a central issue in BR signal transduction. In rice, OsBZR1 positively regulates BR responses, at least in part by repressing BR biosynthetic genes through feedback regulation. Under low-BR conditions, OsGSK2 phosphorylates OsBZR1 and OsLIC, leading to their functional inactivation. Phosphorylated OsBZR1 is exported from the nucleus and retained in the cytoplasm by 14-3-3 proteins, thereby losing its transcriptional regulatory activity. OsLIC acts antagonistically to OsBZR1 by repressing OsBZR1 and ILI1 transcription and by directly activating IBH1 expression. ILI1 and IBH1 also form an antagonistic regulatory pair that controls cell elongation and contributes to regulation of the rice lamina joint angle [55]. OsBSK1-1 has been further identified as a scaffold protein that connects OsBRI1 with OsGSK2. By facilitating this receptor-to-kinase linkage, OsBSK1-1 promotes BR signaling through attenuation of OsGSK2-mediated repression of OsBZR1, thereby establishing an OsBRI1–OsBSK1-1–OsGSK2–OsBZR1 regulatory axis in rice [56] (Figure 2). BR pathways regulate developmental traits, including seed size and grain development. Ubiquitin-mediated regulation contributes to these processes, with rice GW2 acting as an E3 ubiquitin ligase that controls grain size [58,59,60]. 

## 3. Mechanism of Ubiquitination Modification

### 3.1. Ubiquitin–Proteasome System

In eukaryotic cells, the ubiquitin–proteasome system (UPS) mediates selective protein degradation by recognizing ubiquitin-tagged substrates. Beyond protein quality control, the UPS regulates key cellular processes, including cell-cycle progression, DNA replication, chromosome maintenance and hormone signaling. In plant hormone pathways, ubiquitin-mediated protein turnover provides a rapid and reversible mechanism for adjusting signaling intensity, duration and specificity.

In rice, several ubiquitin-related regulators have been implicated in agronomic traits such as grain size, grain weight and plant architecture. For example, GRAIN WIDTH AND WEIGHT 2 (GW2) encodes a RING-type E3 ubiquitin ligase, and loss of GW2 function increases grain width and weight [61]. Other ubiquitin-related regulators, including the GW2–WG1–OsbZIP47 module, the ubiquitin receptor HDR3–GW6a module, OsUBP15 and WIDE AND THICK GRAIN 1/OsTUB1, also influence grain development by modulating protein stability, cell proliferation or cell expansion [62,63,64]. Although these regulators are not all established components of the canonical BR signaling pathway, they illustrate how ubiquitin-mediated regulation can shape crop-relevant developmental traits [65,66,67].

This crop context is relevant to BR biology because BR signaling also regulates grain size, lamina joint inclination, plant architecture and yield-related traits in rice. Thus, rice ubiquitin-related regulators affecting grain development provide a useful conceptual background for understanding how BR-associated ubiquitination modules may be exploited for crop improvement. In the following sections, we therefore focus on ubiquitination and deubiquitination events that are directly linked to BR signaling components, including receptor-level regulation, GSK3-like kinase turnover and BES1/BZR1 stability control.

### 3.2. Ubiquitination Types and Functions in Plants

In plants, ubiquitination, also referred to as ubiquitylation, is a covalent post-translational modification in which ubiquitin is attached to substrate proteins. According to the pattern of ubiquitin attachment, this modification can be classified into monoubiquitination, multi-monoubiquitination and polyubiquitination. Monoubiquitination involves the addition of a single ubiquitin molecule, usually through its C-terminal glycine, to a lysine residue on the substrate, whereas multi-monoubiquitination refers to modification of multiple lysine residues by individual ubiquitin moieties. Polyubiquitination occurs when ubiquitin molecules are linked to one another to form a chain on the substrate. Although polyubiquitination is frequently associated with 26S proteasome-mediated degradation, ubiquitin chains can encode different outcomes depending on their linkage type, length, topology and cellular context.

A central function of ubiquitination is to regulate protein degradation and proteostasis. Misfolded, damaged or overaccumulated proteins are often marked by polyubiquitin chains and removed by the 26S proteasome, thereby maintaining intracellular protein homeostasis [68,69]. Ubiquitination is also widely used in plant hormone signaling. Pathways mediated by auxin, jasmonic acid, gibberellins, abscisic acid and ethylene all depend on ubiquitin-mediated turnover of key receptors, repressors or negative regulators. In these pathways, E3 ubiquitin ligases selectively ubiquitinate specific signaling components, allowing hormone responses to be rapidly activated, dampened or reset [70].

Ubiquitination also regulates many aspects of plant growth and development, including embryogenesis, meristem maintenance, organ size control, floral transition, floral organ formation, seed size determination and seed maturation [71]. In line with this broad role, previous studies and reviews have linked the ubiquitin system to major agronomic traits, including flowering time, seed size and disease resistance. These findings indicate that ubiquitination is not only a protein quality-control mechanism, but also a regulatory system that connects protein homeostasis with hormone signaling, developmental control and crop-relevant traits.

Ubiquitination also provides an important regulatory layer in plant immunity. Immune receptor complexes, downstream kinases and transcriptional regulators are regulated at the levels of activation, trafficking and turnover through ubiquitin-dependent mechanisms [72]. At the receptor level, monoubiquitination and Lys63-linked ubiquitin chains are closely associated with membrane receptor turnover and adjustment of immune signal intensity.

Under abiotic stress conditions, including drought, salinity, heat, cold, oxidative stress and nutrient deprivation, ubiquitination supports rapid proteome remodeling by regulating the stability, activity and localization of transcription factors, transporters and stress-responsive regulators [73]. It also contributes to DNA repair, chromatin regulation and transcriptional control. Lys63-linked chains and selected monoubiquitination events participate in DNA damage responses, whereas histone monoubiquitination can alter chromatin accessibility and gene expression [74].

**Figure 3:**
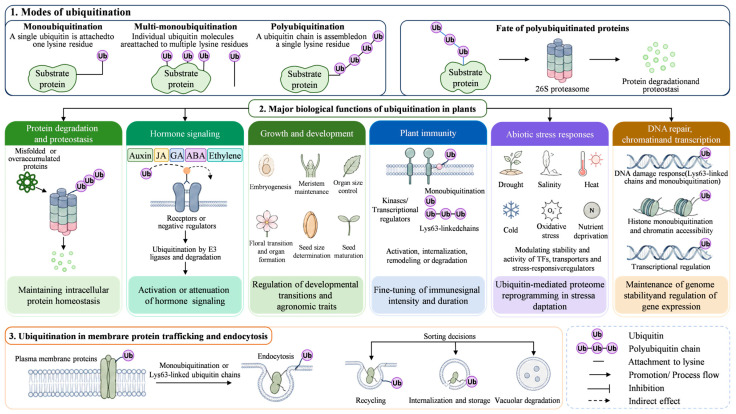
Modes of ubiquitination and biological functions of ubiquitin-mediated regulation in plants. Ubiquitylation 26S proteasome. Beyond proteolysis, ubiquitylation modulates signaling, development, immunity, stress responses, and membrane protein trafficking by controlling protein stability, activity, and localization.

Ubiquitination further regulates membrane protein trafficking and endocytosis. Receptors, transporters and channel proteins at the plant cell surface are often modified by monoubiquitination or Lys63-linked ubiquitin chains, which influence whether they are internalized, recycled to the plasma membrane or targeted to the vacuolar degradation pathway. These examples illustrate how ubiquitination links immune signaling, stress responses, genome maintenance and membrane protein homeostasis through substrate- and context-specific regulatory mechanisms (Figure 3).

### 3.3. E3 Ubiquitin Ligases in Plants

E3 ubiquitin ligases are key enzymes in the ubiquitination cascade and determine much of the substrate specificity of ubiquitin-mediated regulation. Based on their structural organization and catalytic mechanisms, plant E3 ligases can be broadly classified into several major groups, including HECT-type E3 ligases, RING-type E3 ligases, U-box-type E3 ligases and cullin–RING ligases (CRLs).

HECT-type E3 ubiquitin ligases contain a conserved HECT domain of approximately 350 amino acids that mediates ubiquitin transfer. Unlike RING-type E3 ligases, which generally function as scaffolds to facilitate direct ubiquitin transfer from E2 enzymes to substrates, HECT-type E3 ligases form a transient E3–ubiquitin thioester intermediate before transferring ubiquitin to the substrate. Their activity and substrate specificity are influenced by both the cognate E2 ubiquitin-conjugating enzyme and the biochemical properties of the substrate protein. In plants, HECT-type E3 ligases participate in diverse biological processes, including immune responses, growth regulation and developmental control. For example, the Arabidopsis HECT-type E3 ligase UBIQUITIN PROTEIN LIGASE 3 (UPL3) has been implicated in plant immunity and growth regulation [75,76].

RING-type E3 ubiquitin ligases are defined by a conserved RING finger domain, in which cysteine and histidine residues coordinate zinc ions and stabilize the domain structure. Unlike HECT-type E3 ligases, RING-type ligases do not form an E3–ubiquitin thioester intermediate. They instead promote ubiquitin transfer by positioning the E2 ubiquitin-conjugating enzyme close to the substrate, allowing ubiquitin to be transferred directly from the E2 to the target protein. Plants encode a large repertoire of RING-type E3 ubiquitin ligases, many of which are involved in growth, development and stress responses. In *Arabidopsis*, the RING-type E3 ligase SALT- AND DROUGHT-INDUCED RING FINGER 1 (SDIR1) promotes ABA-dependent stomatal closure under drought and salt stress and contributes to drought and salt tolerance [77,78].

U-box-type E3 ubiquitin ligases are characterized by a U-box domain of approximately 70 amino acids. Although this domain is functionally analogous to the RING finger domain, the two domains share no obvious sequence homology. Similar to RING-type E3 ligases, U-box E3 ligases do not form an E3–ubiquitin thioester intermediate. Rather, they position the E2 ubiquitin-conjugating enzyme close to the substrate and promote direct ubiquitin transfer from the E2 to the target protein. Plant U-box proteins (PUBs) form a large family of E3 ubiquitin ligases. In *Arabidopsis*, 64 PUB proteins have been identified, many of which have been implicated in immune signaling, abiotic stress responses, self-incompatibility and hormone signaling [79,80,81,82]. A subset of PUBs regulates ABA-related stress responses. PUB8 targets ABI3 and ABI5 and negatively regulates ABA responses [83]. PUB11 negatively regulates drought tolerance [84], whereas PUB12 and PUB13 modulate ABI1 protein stability in an ABA-dependent manner [85]. PUB18 and PUB19 also attenuate ABA-mediated drought responses [86]. Similarly, PUB22 and PUB23 ubiquitinate the ABA receptor PYL9 and promote its degradation, thereby weakening drought responses [87]. Other PUBs act as positive regulators of stress tolerance. PUB25 and PUB26 promote cold tolerance in *Arabidopsis* by ubiquitinating MYB15, a negative regulator of cold-responsive gene expression, and targeting it for degradation [88]. PUB46 overexpression enhances drought and oxidative stress tolerance in *Arabidopsis* [89]. In wheat (*Triticum aestivum*), TaPUB1 positively regulates salt tolerance [90], whereas TaPUB4 contributes to drought responses [91]. These examples show that PUB proteins can either dampen or enhance stress responses depending on their substrates, upstream signals and physiological context.

Cullin–RING ligases (CRLs) are multisubunit E3 ubiquitin ligase complexes that provide a modular platform for substrate-specific ubiquitination. Cullin proteins act as scaffold subunits, RING finger proteins recruit the E2 ubiquitin-conjugating enzyme, and substrate receptors, including F-box proteins, determine substrate specificity. This architecture allows CRLs to control key regulatory proteins involved in cell-cycle progression, hormone signaling and stress responses. The SKP1–CUL1–F-box (SCF) complex is a major class of CRL-type E3 ligases in *Arabidopsis* and other plants. Through selective ubiquitination and degradation of specific substrates, SCF complexes regulate plant growth, development and adaptation to environmental cues [92].

Importantly, ubiquitination specificity is not determined solely by E3 ligases but also critically depends on E2 ubiquitin-conjugating enzymes, which catalyze ubiquitin transfer from E1 to substrate-bound E3 complexes [79]. Distinct E2 enzymes contribute to the formation of specific ubiquitin chain linkages (e.g., K48- or K63-linked chains), thereby determining whether substrates undergo proteasomal degradation or participate in non-proteolytic regulatory processes [29]. In plants, E2 enzymes can be broadly classified into canonical E2 enzymes, which directly mediate ubiquitin transfer and cooperate with E3 ligases to determine substrate modification and ubiquitin-chain topology, and E2 variants (E2v/E2D-like proteins), which function as accessory factors to facilitate ubiquitin-chain assembly and linkage specificity. Arabidopsis studies have demonstrated that different E2 enzymes participate in developmental regulation and stress responses, highlighting their functional diversification beyond ubiquitin transfer.

Compared with the extensively characterized BR-associated E3 ligases and deubiquitinating enzymes, the specific roles of E2 enzymes in brassinosteroid (BR) signaling remain largely unexplored. Although no BR-specific E2 enzyme has been conclusively identified, key BR signaling components, including the BR receptor BRI1 and downstream regulators BES1/BZR1, undergo ubiquitination-dependent regulation, suggesting that unidentified E2–E3 combinations may determine their ubiquitin-chain architectures and functional outcomes. Therefore, elucidating BR-associated E2 enzymes and their chain-forming properties represents an important direction for understanding ubiquitin-mediated regulation of BR signaling.

## 4. Ubiquitination-Mediated Regulation of BR Signaling

### 4.1. Regulation of BR Signaling at the Receptor Level

At the receptor level of BR signaling, ubiquitination regulates BRASSINOSTEROID INSENSITIVE 1 (BRI1) by controlling its membrane residence, endocytic sorting and degradation. BRI1 is not statically retained at the plasma membrane; instead, it undergoes constitutive trafficking between the plasma membrane, endosomal compartments and the vacuolar degradation pathway. Ubiquitination provides an important sorting signal during this process. Ubiquitinated BRI1 is internalized from the plasma membrane more efficiently and is recognized at the trans-Golgi network/early endosome (TGN/EE), where it is sorted toward vacuolar degradation. By promoting receptor removal from the cell surface and routing BRI1 to terminal degradation, ubiquitination limits prolonged receptor-dependent BR signaling [93].

Further studies identified the plant U-box E3 ubiquitin ligases PUB12 and PUB13 as receptor-associated E3 ligases that catalyse BRI1 ubiquitination. Following BR perception, BRI1 associates with PUB12/PUB13, leading to direct ubiquitination of the receptor. In *pub12 pub13* mutants, BRI1 ubiquitination and endocytosis are markedly reduced, whereas receptor residence at the plasma membrane and total BRI1 abundance are increased. Thus, PUB12/PUB13-mediated ubiquitination promotes BRI1 internalization and subsequent degradation. By limiting the amount of signaling-competent BRI1 at the plasma membrane, PUB12 and PUB13 provide a negative-feedback mechanism that restrains receptor-dependent BR signaling [94].

Concurrently, BRI1 receptor homeostasis is antagonistically regulated by deubiquitination. The deubiquitinating enzymes UBP12 and UBP13 directly interact with BRI1 and remove K63-linked polyubiquitin chains, thereby limiting BRI1 sorting into the vacuolar degradation pathway, maintaining receptor abundance and enhancing plant sensitivity to BRs. Loss of UBP12/UBP13 activity reduces BRI1 protein accumulation and weakens BR responses. Thus, receptor-level control of BR signaling is not a simple one-way ubiquitination-dependent degradation process. Rather, PUB12/PUB13-mediated ubiquitination and UBP12/UBP13-mediated deubiquitination act antagonistically to regulate BRI1 availability at the plasma membrane and adjust BR signaling intensity [95].

### 4.2. Ubiquitin-Mediated Regulation of BIN2

In the BR signaling pathway, BRASSINOSTEROID-INSENSITIVE 2 (BIN2) acts as a central negative regulatory kinase whose stability is controlled by the ubiquitination machinery. This regulation is important for shifting the pathway from a repressed state to an active state. BIN2 is degraded through the 26S proteasome pathway, and the F-box E3 ubiquitin ligase KIB1 is a key mediator of this process. KIB1 interacts directly with BIN2 and promotes its ubiquitination and degradation [96], an effect that is enhanced by BR treatment. Loss of BIN2 accumulation relieves phosphorylation-dependent repression of BES1 and BZR1, allowing these transcription factors to become dephosphorylated, accumulate in the nucleus and activate BR-responsive gene expression [9]. KIB1 also binds BIN2 and disrupts the BIN2–BZR1 interaction, further promoting BR signaling [8]. Thus, ubiquitin-mediated BIN2 degradation functions as a regulatory switch that controls BR signal strength and duration, rather than simply serving as a protein turnover mechanism. This node may also contribute to the adjustment of BR signaling under changing developmental or environmental conditions.

### 4.3. Regulation of the Transcription Factors BES1/BZR1

In the plant BR signaling pathway, ubiquitination regulates BZR1 and BES1 mainly by controlling their stability and directing them to distinct degradation routes. Because these transcription factors act as core downstream effectors, their turnover has a direct impact on the strength and duration of BR transcriptional output. Although BES1/BZR1 mediate activated BR responses, they are not constitutively stable. SINAT E3 ubiquitin ligases can recognize and ubiquitinate BES1/BZR1 and promote their degradation [97].

Under stress, sugar starvation or other growth-limiting conditions, ubiquitinated BES1 can also be recognized by the ubiquitin receptor DSK2 and delivered to the ATG8-associated selective autophagy pathway for degradation [98]. This route attenuates BR-promoted growth and helps plants rebalance growth and survival under adverse conditions [99]. Thus, BES1/BZR1 turnover is not limited to the canonical 26S proteasome pathway but can also occur through ubiquitin-dependent selective autophagy. This provides a mechanistic link between BR signaling, nutrient status and stress-response pathways, with BES1/BZR1 stability serving as a key node for coordinating growth with environmental adaptation.

Meanwhile, deubiquitination provides an opposing mechanism that promotes BES1/BZR1 stability. The deubiquitinating enzymes UBP12 and UBP13 interact directly with BES1 and remove both K48- and K63-linked ubiquitin chains, thereby stabilizing phosphorylated and dephosphorylated BES1 and enhancing plant responsiveness to BRs [96]. Consistently, UBP12 UBP13 loss-of-function plants show reduced BES1 accumulation and BR-defective or BR-insensitive phenotypes, whereas UBP12/UBP13 overexpression enhances BR responses. This regulation is particularly important during recovery from carbon starvation, when induced UBP12/UBP13 expression promotes BES1 re-accumulation and supports renewed growth.

**Figure 4:**
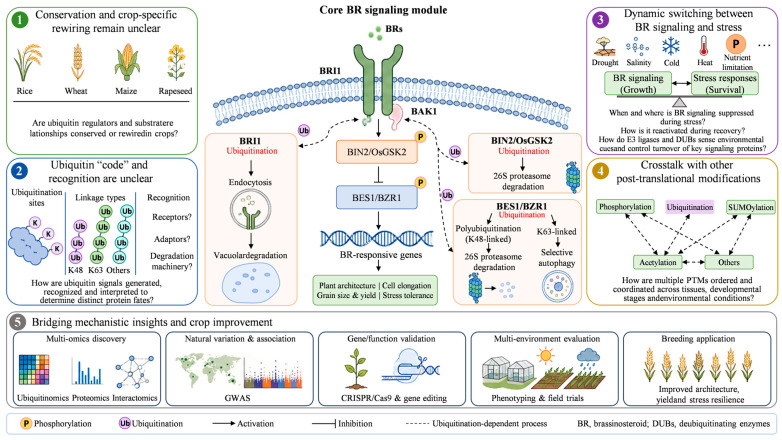
Ubiquitin-dependent control of BES1/BZR1 stability fine-tunes brassinosteroid signaling output. BR signaling promotes BES1/BZR1 accumulation downstream of the BRI1–BAK1 receptor complex. BES1/BZR1 levels are regulated by opposing ubiquitination and deubiquitination pathways. SINATs and BAF1 mediate K48-linked ubiquitination and proteasomal degradation, while DSK2 targets ubiquitinated BES1 for selective autophagy under stress. In contrast, UBP12/UBP13 remove K48/K63-linked ubiquitin chains to stabilize BES1/BZR1 and enhance BR signaling during recovery. Together, these mechanisms maintain BES1/BZR1 homeostasis and regulate BR signaling output.

At the BES1/BZR1 level, BR output is therefore shaped by opposing ubiquitination and deubiquitination activities. SINATs and BAF1 promote BES1 ubiquitination and degradation, whereas DSK2 links ubiquitinated BES1 to selective autophagy under stress or carbon-starvation conditions. In contrast, UBP12/UBP13 stabilize BES1 by removing K48- and K63-linked ubiquitin chains. Together, these opposing mechanisms regulate BES1 abundance and help determine whether BR signaling supports growth or is restrained under metabolic and environmental stress (Figure 4). To better connect Arabidopsis-based mechanistic insights with crop-relevant BR regulation, we summarized the currently known ubiquitination- and deubiquitination-related events affecting BR signaling components in crops and model plants, together with the corresponding E3 ligases, deubiquitinating enzymes and evidence status.

## 5. Roles of Ubiquitination in Plant Growth and Stress Responses

### 5.1. Regulation of Growth and Development

BRs are essential steroid hormones that promote plant growth and regulate developmental processes such as cell elongation, vascular differentiation, root and leaf development, plant height and leaf angle formation. Ubiquitination contributes to the control of BR signaling by regulating the stability, subcellular localization and degradation of key pathway components. Current evidence indicates that the core BR transcription factors BES1 and BZR1 are not constitutively stable; rather, their abundance is continuously adjusted through synthesis, post-translational modification and degradation. Ubiquitination promotes BES1/BZR1 degradation and restricts BR-mediated growth responses, whereas deubiquitination stabilizes BES1 and supports BR-dependent cell elongation and leaf growth. Thus, ubiquitination does not simply turn off BR signaling. By adjusting the abundance of key regulators such as BES1/BZR1, it helps set the level of BR transcriptional output needed for normal plant growth and development [8].

### 5.2. Stress Responses and Hormonal Crosstalk

Under unfavourable conditions such as drought, carbon starvation, salinity and temperature stress, plants transiently restrict growth and reallocate resources toward stress acclimation [100,101,102]. In this context, BR signaling is not simply suppressed but actively remodeled through ubiquitination and related protein turnover pathways. A representative example is BES1, which can be recognized by the ubiquitin receptor DSK2 and directed to ATG8-associated selective autophagy for degradation under stress or energy-limited conditions. This process attenuates BR-mediated growth promotion and facilitates activation of stress-adaptive programs. During recovery or nutrient resupply, deubiquitinating enzymes such as UBP12 and UBP13 stabilize BES1, thereby restoring BR signaling activity and promoting growth resumption.

Beyond stress responses, BR signaling is tightly integrated with multiple hormone pathways, including ABA, auxin, gibberellins (GAs), jasmonates (JAs) and ethylene [103]. Within this network, ubiquitination acts as a central regulatory module that modulates the stability and turnover of shared signaling components, thereby adjusting signaling intensity and response thresholds across pathways. In particular, ubiquitin- and deubiquitinase-mediated control of key regulators such as BES1 provides a mechanistic link between BR signaling, ABA-mediated stress responses and broader hormonal networks [104]. Collectively, ubiquitination functions as a regulatory hub that integrates environmental cues with hormone signaling networks, enabling dynamic balancing between growth promotion and stress adaptation.

## 6. Current Challenges and Future Perspectives

### 6.1. Species Conservation and Divergence of BR Ubiquitination Networks

Although core components of BR signaling, including BRI1, BIN2 and BES1/BZR1, are broadly conserved across plant species, most mechanistic insights into their ubiquitin-mediated regulation have been obtained in Arabidopsis [22,24,96,99]. In contrast, corresponding studies in crops such as rice, wheat, maize and rapeseed remain relatively limited, and whether these regulatory mechanisms are fully conserved or have undergone functional rewiring remains largely unresolved [105,106,107,108].

To provide a systematic overview of the current knowledge on ubiquitination- and deubiquitination-mediated regulation of BR signaling components across model plants and crops, we summarize the available evidence in Table 1.

A major limitation in this field is the lack of systematic functional validation of E3 ligases, deubiquitinating enzymes, and E2–E3 enzyme combinations targeting BR signaling components in crop species. In particular, many regulatory modules identified in Arabidopsis have not yet been confirmed in agronomically important crops, highlighting a substantial knowledge gap between model systems and crop biology. Addressing this challenge will require integrated approaches including CRISPR-based functional genetics, comparative ubiquitinomics, and cross-species complementation analyses.

### 6.2. Ubiquitin Chain Topology and Signaling Specificity

Ubiquitination regulates BR signaling at multiple levels, including receptor turnover (BRI1), kinase stability (BIN2) and transcription factor abundance (BES1/BZR1) [96,97]. However, how ubiquitin chain topology encodes distinct signaling outputs remains insufficiently understood.

While K48-linked chains are generally associated with proteasomal degradation and K63-linked chains with endocytic trafficking and non-proteolytic signaling, the linkage-specific regulation of BR signaling components remains largely unclear [18,27]. In particular, how specific E2–E3 enzyme pairs determine ubiquitin chain specificity in BR pathways is still unknown [30,31]. Resolving this issue will require site-directed mutagenesis of lysine residues, ubiquitin linkage-specific reporters, in vivo ubiquitin chain profiling, and structural characterization of E2–E3-substrate complexes.

### 6.3. Dynamic Coordination Between Growth and Stress Responses

BES1/BZR1 act as central hubs integrating growth and stress responses through ubiquitination and selective autophagy pathways [98,99]. However, how plants dynamically switch between growth-promoting and stress-induced degradation states remains unclear.

Key unresolved questions include how environmental cues modulate E3 ligase and deubiquitinating enzyme activities, how DSK2-mediated selective autophagy is temporally controlled, and how BES1 recovery is achieved following stress release. Addressing these questions will require inducible protein degradation systems, time-resolved proteomics and live-cell imaging approaches under fluctuating environmental conditions [109,110,111].

### 6.4. Integration of Ubiquitination with Other Post-Translational Modifications

BR signaling is regulated not only by ubiquitination but also by phosphorylation, SUMOylation and autophagy-related processes [112,113,114]. These post-translational modifications act in a coordinated and context-dependent manner to control protein stability, localization and activity.

**Table 1:** Cross-species ubiquitination/deubiquitination network regulating BR signaling components in plants.

Function Module	Species	BR Component	E3 Ligase/DUB/Regulator	Regulatory Mechanism	Biological Function	Reference
Receptor regulation	Arabidopsis	BRI1	PUB12/13, ubiquitination machinery	Ubiquitination promotes endocytosis and vacuolar trafficking	Controls receptor turnover and BR sensitivity	[22,94]
Receptor stabilization	Arabidopsis	BRI1	UBP12/UBP13	Deubiquitination stabilizes receptor	Maintains receptor abundance and signaling output	[23]
Kinase control	Arabidopsis	BIN2	KIB1 (F-box E3)	Ubiquitin-mediated degradation of BIN2	Removes BR repressors	[96]
Transcriptional regulation	Arabidopsis	BES1/BZR1	SINAT E3 ligases	Protein ubiquitination and degradation	Controls BR transcriptional output	[97]
Transcriptional regulation	Arabidopsis	BES1	DSK2 (autophagy adaptor)	Selective autophagy-mediated degradation	Balances growth and stress response	[98]
Stress recovery regulation	Arabidopsis	BES1	UBP12/UBP13	Deubiquitination stabilizes BES1	Enhances stress recovery	[24,95]
Rice signaling module	Rice (Oryza sativa)	OsGSK2	TUD1 (U-box E3 ligase)	Ubiquitin-mediated degradation of GSK2	Regulates BR signaling and plant growth	[107]
Rice receptor/growth module	Rice (Oryza sativa)	BR signaling complex	TUD1-Gα module	Regulates signaling output via ubiquitin-associated pathway	Controls plant architecture	[115]
Rice transcriptional module	Rice (Oryza sativa)	OsBZR1	OsPUB24	Ubiquitin-mediated degradation	Regulates BR transcriptional response	[106]
Wheat module	Wheat (Triticum aestivum)	BR signaling components	Putative E3 ligases/DUBs	Unknown mechanistic regulation	BR-growth regulation predicted	[2,111]
Maize module	Maize (Zea mays)	BR signaling components	Putative E3 ligases/DUBs	Unknown mechanistic regulation	BR-related architecture regulation	[2,111]
Rapeseed module	Brassica napus	BR signaling components	Putative E3 ligases/DUBs	Unknown mechanistic regulation	Stress/growth regulation	[2,111]

Increasing evidence suggests a sequential regulatory relationship among these modifications. Phosphorylation may act as a priming event that influences subsequent ubiquitination of key BR components such as BES1/BZR1, thereby modulating their stability and degradation. In parallel, SUMOylation has been proposed to fine-tune ubiquitin-dependent turnover, potentially by altering substrate recognition or competing with ubiquitin modification on shared lysine residues (Table 2).

In core signaling components such as BRI1 and BES1, SUMOylation and ubiquitination likely form a dynamic regulatory switch that balances protein stabilization and degradation, although the precise temporal order of these events remains unclear. Under stress conditions, ubiquitination further connects to selective autophagy, enabling BES1 degradation and attenuation of BR signaling.

Future work integrating multi-PTM proteomics, structural analysis and live-cell imaging will be essential to define the hierarchy and dynamics of these modifications in controlling BR signaling output.

### 6.5. From Mechanistic Insights to Crop Improvement

Despite substantial progress in understanding BR-related ubiquitination mechanisms, their translation into crop improvement remains limited, ubiquitination precisely regulates key agronomic traits through control of receptor abundance, kinase activity and transcription factor stability [115,116].

However, systematic evaluation of BR-related ubiquitin regulators under field conditions is still lacking [117,118]. Future breeding strategies should integrate ubiquitinomics, genome-wide association studies, natural variation analyses and CRISPR-based allele engineering to identify functional variants of E3 ligases, deubiquitinating enzymes and E2 enzymes [119,120].

These candidate regulators must be validated through physiological assays, multi-environment field trials and trait trade-off analyses to ensure agronomic robustness. This pipeline will enable the rational exploitation of BR–ubiquitin regulatory networks for improving crop architecture, yield and stress resilience.

## 7. Conclusions

Brassinosteroid signaling is a central regulatory system governing plant growth, development, and environmental adaptation [1,7]. Substantial progress has elucidated the core signaling cascade from plasma membrane perception to BES1/BZR1-mediated transcriptional regulation [4,5,6,9]. Emerging evidence further demonstrates that ubiquitination and deubiquitination constitute key regulatory modules that fine-tune receptor abundance, kinase activity, and transcription factor stability across the pathway [13,21].

Ubiquitin-mediated regulation provides a dynamic and reversible mechanism to modulate BR signaling output. At the receptor level, BRI1 ubiquitination controls signal initiation and attenuation [22,94], while deubiquitination by UBP12/UBP13 stabilizes receptor abundance [23,95]. At the kinase level, KIB1-mediated degradation of BIN2 acts as a molecular switch for pathway activation [96]. At the transcriptional level, BES1/BZR1 stability is regulated by E3 ligases such as SINATs and BAF1, as well as selective autophagy mediated by DSK2 [97,98,99], enabling rapid adjustment to developmental and environmental cues.

Importantly, ubiquitination operates within an integrated post-translational modification (PTM) network, where phosphorylation, SUMOylation, and autophagy coordinately regulate substrate fate and signaling dynamics [112,113,114]. This multilayered crosstalk supports the concept that BR signaling is governed by a dynamic regulatory code rather than isolated modification events. Despite recent advances, key questions remain regarding conservation across crop species, ubiquitin chain topology and specificity, and PTM hierarchy under environmental fluctuations [2,18,27].

From an applied perspective, integrating ubiquitin biology with crop genetics provides a promising strategy for improving plant architecture, yield, and stress resilience [3,105,111]. The BR–ubiquitin regulatory network therefore represents a valuable and largely untapped resource for precision breeding in future agriculture [33,121].

## Figures and Tables

**Table 2:** Key unresolved questions and experimental strategies in the BR–ubiquitin signaling network.

Regulatory Level	Challenge	Key Mechanistic Question	Experimental Approaches
Receptor level	Receptor trafficking	Is ubiquitin-mediated endocytosis of BRI1 conserved across crop species?	CRISPR; localization assays; ubiquitin profiling
Kinase level	Negative regulation	E3 ligases regulating GSK3-like kinases in crops?	Genetic screening; Co-IP/MS; CRISPR functional validation
Transcriptional level	TF stability	BES1/BZR1 regulation by E3 ligases and DUBs?	Ubiquitinome profiling; protein stability assays; Co-IP/MS
Transcriptional level	Stress integration	Selective regulation of BES1/BZR1 under stress and growth conditions?	Inducible stress; autophagy mutants; live imaging
PTM crosstalk	Modification hierarchy	How do phosphorylation, SUMOylation, and ubiquitination coordinate BR signaling output?	Multi-PTM proteomics; site-directed mutagenesis (lysine residues); structural biology
Cross-species conservation	Evolutionary conservation	Are Arabidopsis-defined ubiquitin regulatory modules conserved in rice, wheat, maize, and rapeseed?	Cross-species complementation; comparative ubiquitinomics; CRISPR in crops
Crop gap	Functional validation	Which BR-related ubiquitin regulators remain uncharacterized in crops?	Pan-genome mining; mutant screening; genomics

## Data Availability

Data availability is not applicable to this article as no datasets were generated or analyzed during the current study.
